# Twenty-four-week safety and tolerability of nevirapine *vs.* abacavir in combination with zidovudine/lamivudine as first-line antiretroviral therapy: a randomized double-blind trial (NORA)

**DOI:** 10.1111/j.1365-3156.2007.01973.x

**Published:** 2008-01

**Authors:** Dart Trial Team

**Keywords:** antiretroviral therapy, Africa, randomized-controlled trial, HIV, toxicity

## Abstract

**Objective:**

To compare the safety/tolerability of abacavir and nevirapine in HIV-infected adults starting antiretroviral (ARV) therapy in Uganda.

**Methods:**

Twenty-four-week randomized double-blind trial conducted with 600 symptomatic ARV-naive adults with CD4 <200 cells/mm^3^ allocated to zidovudine/lamivudine plus 300 mg abacavir (A) and nevirapine placebo (*n* = 300) or 200 mg nevirapine (N) and abacavir placebo (*n* = 300) twice daily. The primary endpoint was any serious adverse event (SAE) definitely/probably or uncertain whether related to blinded nevirapine/abacavir. Secondary endpoints were adverse events leading to permanent discontinuation of blinded nevirapine/abacavir, and grade 4 events.

**Results:**

Seventy-two per cent participants were women; 19% had WHO stage 4 disease; the median age was 37 years (range 18–66); the median baseline CD4 count was 99 cells/mm^3^ (1–199). Ninety-five per cent completed 24 weeks: 4% died and 1% were lost to follow-up. Thirty-seven SAEs occurred on blinded drug in 36 participants. Twenty events [6 (2.0%) abacavir, 14 (4.7%) nevirapine participants] were considered serious adverse reactions definitely/probably/uncertain whether related to blinded abacavir/nevirapine [HR = 0.42 (95% CI 0.16–1.09) *P* = 0.06]. Only 2.0% of abacavir participants [six patients (0.7–4.3%)] experienced a suspected hypersensitivity reaction (HSR). In total 14 (4.7%) abacavir and 30 (10.0%) nevirapine participants discontinued blinded abacavir/nevirapine (*P* = 0.02): because of toxicity (6A, 15N; *P* = 0.07, all rash/possible HSR and/or hepatotoxicity), anti-tuberculosis therapy (6A, 13N), or for other reasons (2A, 2N).

**Conclusions:**

There was a trend towards a lower rate of serious adverse reactions in Ugandan adults with low CD4 starting ARV regimens with abacavir than with nevirapine. This suggests that abacavir could be used more widely in resource-limited settings without major safety concerns.

## Introduction

The first-line regimen recommended by WHO in resource-limited settings is two nucleoside/nucleotide reverse transcriptase inhibitors (NRTIs) plus a non-nucleoside reverse transcriptase inhibitor (NNRTI) (WHO 2006). Nevirapine has been the most frequently used NNRTI, primarily because of low cost and incorporation into fixed dose combinations: the alternative NNRTI, efavirenz, is contraindicated in pregnancy and is therefore problematic in settings with a high proportion of women of child-bearing potential. Rash and hepatoxicity are the most common toxicities associated with nevirapine. Rashes occur in 15–20% of subjects (de Maat *et al.* 2003; Montaner *et al.* 2003). They are severe, life-threatening or fatal in 2–5% subjects (van Leeuwen *et al.* 2003), and cause discontinuation in around 7% (Knobel *et al.* 2004; van Leth *et al.* 2004). First-line therapy with 2NRTI/NNRTI is limited by interactions between NNRTIs and anti-tuberculosis treatment; hepatotoxicity of nevirapine in those with higher CD4 counts [particularly women (van Leth *et al.* 2005) for whom efavirenz is contraindicated if they wish to become pregnant] or those co-infected with hepatitis C.

Triple NRTI regimens have potential advantages over standard NNRTI-based first-line regimens in Africa, as they avoid drug interactions with TB therapy, can be taken by women who may become pregnant and those with higher CD4 counts, consist of fewer pills and spare two classes for second-line after immunological/clinical failure where drug resistance is likely. Whilst it is generally recognized that triple NRTI regimens have poorer virological efficacy than NNRTI or protease inhibitor (PI)-based therapy (Gulick *et al.* 2004; Bartlett *et al.* 2006) a ‘simplification strategy’ for managing NNRTI toxicity and drug–drug interactions by substituting with a third NRTI, either abacavir or tenofovir, is also evolving (Gilks *et al.* 2006). Abacavir is also an important potential backbone NRTI combined with NNRTIs (WHO 2006). However, 3–8% of patients receiving abacavir in clinical studies in industrialized countries develop a suspected HSR (Brothers *et al.* 2005), characterized by fever, rash, gastrointestinal and/or respiratory symptoms, and lethargy or malaise, which usually appear within 6 weeks. Symptoms worsen with continued therapy but usually resolve on discontinuation. Restarting abacavir results in a prompt return of symptoms, which may be more severe and include life-threatening hypotension and death. Standard practice in industrialized countries is to stop abacavir for symptoms consistent with hypersensitivity and never restart the drug. Although genetic variations in HLA-B have been associated with abacavir HSRs in some populations (Hughes *et al.* 2004), such tests are unlikely to be available throughout Africa to guide its use. Thus whilst rates of abacavir HSR are likely to be lower in Africa than in industrialized countries as a result of race and lower pre-ART CD4 (Brothers *et al.* 2006), high rates of possible reactions that cannot be confirmed or may be confused with malaria or other infections or immune reconstitution disease could render abacavir challenging to use in Africa, or substantially reduce its use.

Nevirapine OR Abacavir (NORA) was therefore designed to evaluate the safety of abacavir compared with nevirapine in previously untreated African individuals with advanced HIV disease initiating ARVs in a setting where all patients were under close clinical supervision within the DART trial.

## Methods

### Trial design

Nevirapine OR Abacavir was a 24-week randomized double-blind trial conducted in two centres in Uganda (the Joint Clinical Research Centre, Kampala and the MRC/UVRI Uganda Research Unit on AIDS, Entebbe, Uganda), as a nested substudy within DART (Reid *et al.* 2004). NORA participants were randomly allocated in a 1:1 ratio to receive zidovudine and lamivudine (co-formulated as combivir) plus either 300 mg abacavir and nevirapine placebo or abacavir placebo and 200 mg nevirapine twice daily prescribed for 24 weeks (double dummy design). The dose of blinded nevirapine or placebo was doubled from 200 mg once daily to twice daily at 14 days. Participants experiencing adverse events considered suspected reactions to abacavir or nevirapine after discussion with the Project Leader in each site were to be unblinded (after completion of case record forms) to avoid the possibility of restarting abacavir or nevirapine in a participant experiencing a drug-related reaction. Other participants needing to switch from blinded nevirapine/abacavir (e.g. to start anti-tuberculosis medications) were to substitute tenofovir DF without unblinding. After 24 weeks, participants continued combivir, substituting open-label nevirapine or abacavir for active trial drug and continued follow-up in DART.

### Participants and sites

Symptomatic HIV-antibody positive adults aged 18 years or older with CD4 <200 cells/mm^3^ who had not previously received ARVs other than to prevent mother-to-child HIV transmission and without concurrent acute infections who were being enrolled into the DART trial in Uganda were eligible for NORA unless they had laboratory abnormalities contraindicating nevirapine or were taking tuberculosis treatment (intensive or maintenance phase). Every participant gave informed consent for both NORA and DART, which received ethics approval in Uganda and the UK. Staff in both sites received standard training in abacavir hypersensitivity recognition and management prior to the trial using a standard case definition (Brothers *et al.* 2005). In addition, all NORA participants in NORA were issued with an ‘Abacavir Warning Card’.

### Randomization

Randomization was undertaken by telephoning the local Trials Centre attached to but separate from each of the clinical sites, stratified by clinical centre, baseline CD4 count (0–99 or 100–199 cells/mm^3^) and allocation to clinical monitoring only (CMO) or laboratory plus clinical monitoring (LCM) in the main DART trial. Blinded trial drug was labelled with sequential study numbers according to a pre-prepared randomization list generated by the Trial Statistician using simple randomization within strata. Randomization codes linking active nevirapine/abacavir allocation to study number (and thus to blinded labelled drug) were only held by one statistician at each local Trial Centre (who could therefore perform emergency unblinding) and two statisticians at the coordinating centre in London. Thus neither the patient, the treating physician, data entry staff nor staff at the local Trials Centre knew which arm the participant was allocated to.

### Management and follow-up

Trial entry was the date of randomization. All DART participants attend the study clinic 4 weekly, when a nurse administers a standard symptom checklist and adherence questionnaire, and dispenses a repeat ARV prescription (28 days); participants are also asked to return if they feel unwell. All participants see a doctor and have a full blood count, liver and renal function tests, and lymphocyte subsets measured at weeks 4 and 12, and then every 12 weeks. However, the laboratory results are *only* returned to clinicians caring for CMO participants in case of grade 4 toxicity or if clinically indicated and lymphocyte subsets are not returned. Laboratory tests may be requested for clinical indications at any time. Serious adverse events (SAEs) according to the International Conference on Harmonization Good Clinical Practice (ICH-GCP) and new/recurrent WHO stage 3 and 4 events (WHO 1990) are reported at diagnosis: all other events are reported at routine follow-up visits. All WHO stage 4 events are reviewed against criteria for presumptive/definitive diagnosis by an Endpoint Review Committee (blinded to allocated treatment), who also review cause of death. All reported SAEs in NORA were also reviewed blinded to allocated treatment. Adherence was assessed by pill counts at 4-weekly nurse visits, adjusting for late return to clinic (in 1–3%), early attendance or dose escalation, but not adjusting for periods off ARVs or for additional pills prescribed if a patient knew they could not attend their next visit in exactly 28 days.

### Outcomes

The primary endpoint was any SAE which was definitely/probably related or uncertain whether related to blinded nevirapine/abacavir (serious adverse reaction – SAR) occurring while on ARVs or within 30 days of stopping ARVs. The original sample size of 600 provided 80% power to detect a difference in rate of SARs between 15% in one and 8% in the other arm using a two-sided chi-squared test with α = 0.05. Following ICH guidelines, SAEs were defined as events not related to HIV *only* and fatal, life-threatening, causing unplanned or prolonging hospitalization, causing permanent or significant disability, or other important medical conditions. All suspected HSRs (which carry a risk of life-threatening symptoms without drug discontinuation) were considered ‘other important medical conditions’. Secondary endpoints were adverse events of any grade leading to permanent stopping of blinded nevirapine/abacavir, and grade 4 events irrespective of whether or not they resulted in stopping nevirapine/abacavir [toxicity graded accorded to minor modification of the AIDS Clinical Trials Group (ACTG) criteria (Division of AIDS 1992)]. There was no requirement to report laboratory or clinical grade 4 AEs as SAEs unless they met the ICH-GCP criteria.

### Statistical analysis

Time-to-event methods including Kaplan–Meier plots, logrank test and Cox proportional hazards models were used to compare randomized groups for time-to-event outcomes. Categorical variables were compared between randomized arms using exact tests, and continuous variables using *t*-tests and rank-sum tests. All comparisons were as randomized (intent to treat): safety analyses were restricted to time on blinded nevirapine/abacavir plus 30 days (similar results including all follow-up). Baseline values were those recorded nearest to but before and within 6 weeks of randomization. Generalized estimating equations (GEE, independent correlation structure) were used to compare continuous variables across randomized groups over time, using the closest measurement to the scheduled assessment week within equally spaced windows. All *P*-values are two sided. All analyses were repeated with and without stratification for baseline CD4 cell count, centre and randomization to CMO *vs.* LCM to confirm no major imbalances affecting results.

## Results

**Figure 1:**
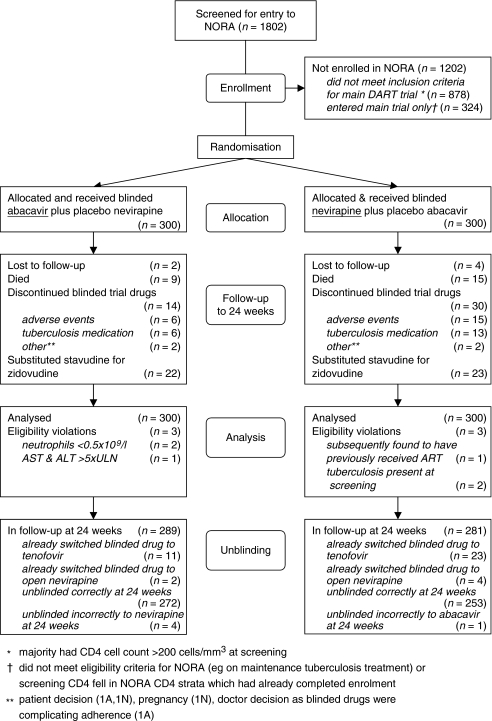
Participant disposition.

Between 5 January 2004 and 28 October 2004, 600 individuals were randomized (Figure 1). A year after enrolment, one participant (N) disclosed that they had previously taken 3 months of Triomune (co-formulated lamivudine/stavudine/nevirapine). As the number of other participants with concealed prior exposure is unknown, this participant was not excluded. Two further participants (2N) had tuberculosis at screening: one concealed a recent diagnosis from fear of not receiving ART, and one was diagnosed after randomization from a screening sputum sample; a further three participants (3A) had minor eligibility violations (two neutrophils <0.5 × 10^9^/l, 1 ALT/AST >5 × ULN). Primary and secondary endpoint comparisons were identical if restricted to the strictly eligible population (297A, 297N). All but one participant started allocated trial drugs within 3 days of randomization; this last participant started on day 8.

### Baseline characteristics

Baseline characteristics were broadly similar between the two groups (Table 1). Fourteen of the 15 women who reported previous ARVs to prevent mother-to-child HIV transmission had taken single-dose nevirapine: the remaining woman did not know which drug(s) she had received. One hundred and twenty-five (42%) abacavir and 120 (40%) nevirapine participants were taking cotrimoxazole prophylaxis or started it at randomization (median CD4 104 cells/mm^3^): only 11 (2%) and one (0.2%) participants were taking fluconazole or isoniazid prophylaxis, respectively.

**Table 1:** Baseline characteristics

	Abacavir	Nevirapine
Total participants	300 (100%)	300 (100%)
Randomization in main DART trial		
Laboratory and clinical monitoring	150 (50%)	149 (50%)
Clinical monitoring only	150 (50%)	151 (50%)
Centre		
Entebbe, Uganda	149 (50%)	151 (50%)
JCRC, Uganda	151 (50%)	149 (50%)
Sex		
Male	83 (28%)	87 (29%)
Female	217 (72%)	213 (71%)
Age (years)		
≤30	43 (14%)	68 (23%)
>30–35	69 (23%)	55 (18%)
>35–40	75 (25%)	90 (30%)
>40–45	64 (21%)	41 (14%)
>45–50	31 (10%)	32 (11%)
>50	18 (6%)	14 (5%)
Median (IQR)	37.6 (31.9–42.3)	36.3 (30.7–41.4)
WHO disease stage		
2	83 (28%)	76 (25%)
3	173 (58%)	157 (52%)
4	44 (15%)	67 (22%)
CD4 (cells/mm^3^)		
0–49	76 (25%)	88 (29%)
50–99	75 (25%)	62 (21%)
100–149	75 (25%)	84 (28%)
150–199	74 (25%)	66 (22%)
Median (IQR) [range]	99 (49–149) [1–199]	100 (40–145) [1–199]
Body mass index (BMI): median (IQR)	20.9 (18.9–23.5)	21.3 (19.3–23.8)
Haemoglobin (g/dl): mean (SD)	11.5 (1.8)	11.6 (1.7)
Neutrophils (×10^9^/l): mean (SD)	1.5 (0.7)	1.6 (0.9)
Glomerular filtration rate (ml/min/1.73 m^2^)*: mean (SD)	85 (27)	85 (20)
ALT (IU): mean (SD)	27 (19)	27 (16)
AST (IU): mean (SD)	37 (25)	37 (20)
Women prescribed ARVs to prevent MTCT before entering DART (% of women)	4 (2%)	11 (5%)
Cotrimoxazole prophylaxis†	120 (40%)	125 (42%)

The median CD4 at baseline is 99 rather than 99.5 cells/mm^3^ (as designed), because one participant was randomized in the wrong CD4 stratum in error.

ARV, antiretroviral; WHO, world health organisation.

* Calculated according to the Cockcroft–Gault formula and adjusted for body surface area.

† Including prophylaxis prescribed on the day of randomization.

### Follow-up

Five hundred and seventy (95%) participants (289A, 281N) completed 24 weeks: 24 participants died before 24 weeks (9A, 15N), five were lost to follow-up (1A, 4N) and one (A) formally withdrew consent (Figure 1). Participants reaching week 24 were transferred to open-label nevirapine or abacavir and continue to be followed-up in DART.

### Adherence

There was no difference between randomized groups in the proportion with >95% adherence according to 4-weekly pill counts to combivir (global *P* = 0.84), blinded abacavir/abacavir placebo (*P* = 0.60) or blinded nevirapine/nevirapine placebo (*P* = 0.96). In both groups and for all drugs, the proportion reporting >95% adherence increased over time, from 85% of participants at week 4 to 92% at week 12 and 94% at week 24. However, the percentage of participants reporting that they had never missed a dose decreased from 73% at 4 weeks to 61% and 49% at 12 and 24 weeks with no difference between arms (global *P* = 0.58), although the percentage reporting that they had missed a dose in the last week remained stable at 4–6%, again with no difference between arms (global *P* = 0.55).

### Serious adverse reactions (SARs, primary endpoint)

**Figure 2:**
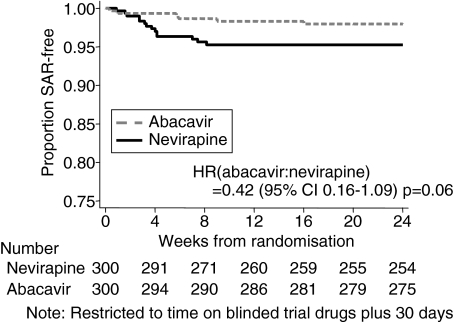
Time to first serious adverse reaction (serious adverse event definitely/probably related or uncertain whether related to blinded trial drugs).

A total of 37 SAEs occurred in 36 participants (14A, 22N; a second SAE occurred in one participant on open-label nevirapine following discontinuation of blinded abacavir for HSR), all on blinded drug or within 30 days of stopping blinded trial drugs. Twenty (6A, 14N) were considered to be SARs at independent review (definitely/probably related or uncertain whether related to blinded trial drugs, Table 2) occurring in six (2.0%) and 14 (4.7%) participants receiving abacavir and nevirapine respectively [HR = 0.42 (95% CI 0.16–1.09), logrank *P* = 0.06, Figure 2]. The 20 SARs were one life-threatening event (N), two hospitalizations (2N), and 17 other important medical conditions (6A, 11N); only 10 (2A, 8N) were grade 3/4. Three (3N) and one (N) SAR were also considered uncertain whether related to cotrimoxazole and isoniazid respectively. The six abacavir SARs were in three men and three women, whereas the 14 nevirapine SARs were in three men and 11 women (*P* = 0.19 for heterogeneity with sex). Seven additional SAEs (3A, 4N) were originally reported as uncertain whether related to blinded trial drugs by the clinical investigator, but were judged unlikely to be related at independent review (Table 2).

**Table 2:** Grade 4 and serious AEs and ARs

	Abacavir		Nevirapine	
		
	Events	Reactions*	Events	Reactions*
**Total SAE/SAR**	**15**	**6 [3]****†**	**22**	**14 [4]****†**
Grade 4	7	[2]	11	5 [2]
Suspected HSR			4	4
Acute asymptomatic hepatitis (LFTs)			1	1
Anaemia	4		3	[1]
Pancytopenia + haematemesis	1	[1]		
Sepsis + haematemesis			1	[1]
Deep vein thrombosis	1	[1]		
Head injury			1	
Indeterminate cerebral disease			1	
Urticarial rash + fever + mucosal symptoms	1§			
Grade 1–3	7	6	10	9 [1]
Suspected HSR	6	6	9	9
Duodenal ulcer + haematemesis	1			
Fever + constitutional symptoms			1¶	[1¶]
Death from unknown cause	1	[1]	1	[1]
Participants with at least one SAE/SAR (% of randomized‡)	14 (5%)	6 (2%)	22 (7%)	14 (5%)
Rate (events per 100 person years)	11.4	4.6	17.7	11.2
**Grade 4 AE/AR not reported as SAE/SAR (not reviewed)**	**73**	**[11]**	**105**	**[11]**
Anaemia (<6.5 g/dl)	14		13	
Neutropenia (<0.5 × 10^9^/l)	47	[9]	73	[11]
Leucopenia (<1.0 × 10^9^/l)	5		5	
Thrombocytopenia (<20 × 10^9^/l)	1		1	
Raised bilirubin (>5.0 × ULN)	2		3	
Raised creatinine (>6.0 × ULN)			1	
Raised liver enzymes (>10.0 × ULN)			7	
Hyponatraemia (<116 mmol/l)	1		1	
Myopathy	1	(1)		
Paralytic ileus			1	
Convulsions	2	(1)		
Participants with at least one grade 4 AE/ARnot reported as SAE/SAR (% of randomized‡**)	61 (20%)	11 (4%)	92 (31%)	11 (4%)
**Total grade 4 AE/AR (including grade 4 SAE/SAR)**	**80**	**13**	**116**	**18**
Rate (events per 100 PY)	60.7	9.9	93.2	14.5
Participants with at least one grade 4 AE/AR (% of randomized‡)	65 (22%)	13 (4%)	98 (32%)	18 (6%)

Grade 4 AE are new grade 4 AE reported during the first 24 weeks which were not pre-existing at randomization and excluding recurrences of the same event. Grade 4 laboratory results are returned to clinicians (in both LCM and CMO arms, see *Methods*), but there was no requirement to report a clinical or laboratory grade 4 AE as an SAE unless the event met the ICH-GCP criteria for SAE.

SAE, serious adverse event; SAR, serious adverse reaction; HSR, hypersensitivity reaction.

* Definitely/probably related or uncertain whether related to blinded trial drugs.

† For SAE: number of confirmed SARs at independent SAE review [additional SAE originally reported by the clinical investigator as uncertain whether related to blinded trial drugs, but judged unlikely to be related or unrelated to blinded trial drugs at independent review]. For grade 4 AE (not reviewed): [any grade 4 event reported by the clinical investigator as definitely/probably related or uncertain whether related to blinded trial drugs].

‡ 600 randomized, 300 to abacavir and 300 to nevirapine.

§ Related to open label nevirapine following discontinuation of blinded abacavir for a previous HSR.

¶ Considered uncertain whether related to rabies vaccination on independent review but unlikely to be related to blinded trial drug.

** Participants with SAE reported could have laboratory Grade 4 AE at the same time which did not meet ICH-GCP SAE criteria.

In 19 of 20 participants, the SAR was consistent with a suspected HSR: six abacavir (2.0%, 95% CI 0.7–4.3%) *vs.* 13 nevirapine (4.3%, 95% CI 2.3–7.3%). Broadly similar proportions of those with suspected HSR on abacavir and nevirapine had fever (4/6A, 9/13N); respiratory (4A, 7N), constitutional (3A, 7N), or gastrointestinal (2A, 5N) symptoms; rash (6A, 10N) and oral/mucosal involvement (3A, 6N): but hepatic involvement was only seen in the nevirapine group (4N) (Table 3). All participants with respiratory symptoms in the nevirapine group had either rash (*n* = 4) or hepatic involvement plus constitutional symptoms and fever (*n* = 3). Fifteen of the 16 rashes were disseminated; three were grade 4 (3N), one grade 3 (A) and 11 (5A, 6N) grade 1/2. No participant had Stevens–Johnson syndrome. The remaining SAR was asymptomatic grade 4 hepatitis (N). In total, five of the 14 nevirapine participants with SARs had grade 4 LFT elevations (including the one with asymptomatic hepatitis alone) and two had grade 3 LFTs, compared with none of the six abacavir participants.

**Table 3:** Symptoms occurring in participants with suspected HSRs

	Total	Abacavir	Nevirapine
Suspected HSRs	19	6	13
Predominant symptomcomplex:			
Rash, erythematous, multiforme	1	0	1
Rash, maculopapular and/orerythematous			
Rash alone	2	1	1
+ Constitutional symptoms only	3	1	2
+ Mucosal symptoms only	5	2	3
+ Mucosal + constitutionalsymptoms	2	0	2
+ Mucosal symptoms + hepatitis	1	0	1
Rash, urticarial			
+ Constitutional symptoms only	1	1	0
+ Mucosal + constitutionalsymptoms	1	1	0
Acute hepatitis + constitutionalsymptoms	3	0	3
Individual skin components*:	16	6	10
Pruritis	11	4	7
Urticaria	5	3	2
Erythema multiforme	2	1	1
Mucous membrane involvement	4	1	3
Macular/maculopapular rash	11	3	8
Vesicular	1	1	0
Erythema	6	3	3
Other symptom components*			
Fever/chills	13	4	9
Malaise/fatigue	7	2	5
Myalgia/arthralgia	4	0	4
Headache	7	3	4
Conjunctivitis	3	3	0
Stomatitis	4	0	4
Other mucosal lesions	2	0	2
Nausea/vomiting	4	0	4
Diarrhoea	1	1	0
Abdominal pain	4	1	3
Cough/pharyngitis	11	4	7
Dyspnoea/wheezing	0	0	0
Oedema	5	1	4
Tachycardia	2	0	2
Hypotension	1	1	0
Other	2	1†	1‡
LFT toxicity			
Grade 4	4§	0	4§
Grade 3	2	0	2

HSR, hypersensitivity reaction.

* Each component present (to any degree) or not present in the reaction. Therefore each participant may report multiple symptoms, in contrast to ‘predominant symptom complex’ where each participant appears only once.

† Burning sensation to both feet.

‡ Jaundice.

§ One additional SAR (asymptomatic hepatitis) also had grade 4 LFT elevations.

The 17 (9A, 8N) SAEs considered unlikely to be related to blinded drug were anaemia (*n* = 7, one fatal), pancytopenia/haematemesis (one fatal), sepsis/haematemesis (one fatal), death from sudden/unexpected cause/death at home [two at weeks 4 (N) and 9 (A), neither with any recorded interruption in blinded drugs], indeterminate cerebral disease (fatal), head injury, DVT, duodenal ulcer/haematemesis, fever (uncertain whether related to rabies vaccination), and rash (related to open label nevirapine following discontinuation of blinded abacavir for a previous SAR) (Table 2).

### Grade 4 adverse events

There were significantly more grade 4 AE in the nevirapine than in the abacavir arm [98 (33%) *vs.* 65 (22%) participants, rates 93 and 61 per 100 person years, *P* = 0.003]. The majority were neutropenia (47A, 73N) or anaemia (19A, 15N). Only nine participants (9N) ever had grade 4 (>10 × upper limit of normal) elevations in LFTs (including one with asymptomatic hepatitis and four with suspected HSR reported as SARs). Four resolved after substitution of tenofovir for nevirapine (all four reported as SARs), and five resolved without substituting for nevirapine (4 had one grade 4 measurement only, the remaining one (reported as a SAR) was maintained on open-label nevirapine). Of the 196 grade 4 AE, only 31 (13A, 18N) were considered by the clinical investigator to be definitely/probably or uncertain whether related to blinded trial drugs [in 13 (4%) *vs.* 18 (6%) participants, respectively], with no evidence of a difference between randomized groups (rates 9.9 and 14.5 per 100 person-years, *P* = 0.30).

### Discontinuation of blinded trial drug

Blinded trial drug was prescribed for 96.8% and 92.3% of person-time to 24 weeks in the abacavir and nevirapine arms, respectively. In total 14 (4.7%) and 30 (10.0%) participants discontinued blinded trial drug, respectively (exact *P* = 0.02, *P* = 0.04 in strictly eligible population). Twenty-one discontinuations were for toxicity [6A (2.0%) *vs.* 15N (5.0%), exact *P* = 0.07] and included all 20 participants with SARs: the toxicity was rash/suspected HSR (6A, 13N) and/or hepatotoxicity (4N) in all cases. The remaining 19 patients were to start tuberculosis therapy [6A (2.0%) *vs.* 13N (4.3%), respectively], or for other reasons [pregnancy (1N), personal (2A, 1N)]. However, two of the 13 discontinuing blinded trial drug to start tuberculosis therapy in the nevirapine arm had tuberculosis which could have been identified at screening. Time to AE causing discontinuation did not differ significantly between the two groups [median 32 days (IQR 25–59, range 8–85) in the nevirapine *vs.* 44 days (12–66, 4–112) in the abacavir group; ranksum *P* = 0.70].

### Unblinding

Twenty-two participants (6A, 16N) were unblinded before 24 weeks – all but one (N, pregnancy) because of toxicity leading to discontinuation of blinded trial drugs described above. Sixteen (4A, 12N) of the 22 substituted tenofovir according to protocol and six (2A, 4N) substituted open-label nevirapine. Of the four participants who therefore remained on active nevirapine, one unblinded for pregnancy, one for fever/constitutional symptoms subsequently judged not related to blinded trial drugs and uncertain whether related to rabies vaccine (Table 2), and two for suspected HSRs (grade 1 and 2 reactions, respectively).

### Other substitutions and discontinuations whilst retaining blinding

A further 20 (7A, 13N) participants substituted open-label tenofovir for blinded trial drugs without unblinding (most following tuberculosis diagnosis). Only two participants (1A, 1N) stopped all antiretrovirals for >31 days (both for personal reasons). Forty-five participants (22A, 23N) substituted stavudine for zidovudine, because of anaemia (16A, 17N) and/or neutropenia (5A, 8N), or myopathy (1A). No other substitutions occurred during the 24-week study, and no participant switched to second-line therapy.

### Changes in laboratory parameters of toxicity

The nevirapine arm had significantly greater increases from baseline in absolute levels of liver function tests (ALT global *P* = 0.003, AST *P* = 0.07) compared with the abacavir arm, and significantly higher grades of liver function test toxicity (ALT *P* = 0.006, AST *P* = 0.05). In contrast, the nevirapine arm had small but significantly greater increases from baseline in glomerular filtration rate calculated by Cockcroft–Gault (GFR) compared with the abacavir arm (global *P* = 0.01, e.g. mean +1 *vs.* +9 in the nevirapine arm at week 12, *t*-test *P* = 0.007), and lower grades of GFR toxicity (*P* = 0.03). Early haemoglobin decreases were smaller in the abacavir arm (mean −0.3 g/dl *vs.*−0.6 g/dl in the nevirapine arm at week 4, *t*-test *P* = 0.02) although comparable by week 24 (global *P* = 0.09) and there was no overall difference in toxicity (*P* = 0.90). Similarly, there was a non-significant trend towards smaller decreases in neutrophils in the abacavir arm (*P* = 0.43), and significantly lower grades of toxicity (*P* = 0.03). There was no difference between nevirapine and abacavir arms in other laboratory parameters of toxicity.

## Discussion

This is the first blinded study to compare reactions to abacavir and nevirapine. The low rate recorded for abacavir (2.0%, 95% CI 0.7–4.3%) demonstrates that the potential for HSRs should not preclude the use of this drug in Africa and other resource-limited settings rolling out ART. In particular, in spite of the higher background rates of intercurrent infection and malaria, the only abacavir discontinuations for toxicity were for HSRs confirmed as serious adverse reactions at independent review. Clinicians in DART did not have any previous experience of using abacavir, showing that with focussed HSR training, clinical teams in resource-limited settings can identify and manage abacavir reactions appropriately. In addition, none of the six abacavir reactions were grade 4, suggesting that patients in these settings can present promptly to clinical services with suspected HSR symptoms if this is explained well. By comparison, 4.7% (95% CI 2.6–7.7%) of the nevirapine group developed serious adverse reactions, and four of the 13 suspected HSR in participants receiving nevirapine were grade 4.

Nevirapine OR Abacavir is the first trial to compare all adverse reactions between abacavir and nevirapine using blinded drugs. Results indicate a higher level of discontinuation of nevirapine than of abacavir overall and a trend towards a higher rate of serious adverse reactions and discontinuation for toxicity with nevirapine. Nevirapine is widely used in resource-limited settings, but has the potential for life-threatening reactions. These data suggest that, based on toxicity profile, abacavir could be used more widely in resource-limited settings without major safety concerns. We observed considerable overlap in the clinical manifestations of nevirapine and abacavir related reactions, noting however, that hepatotoxicity only occurred with nevirapine. These data suggest that whilst the use of abacavir and nevirapine in combination is attractive (particularly with lamivudine as part of the fixed dose combination, Kivexa), strategies for managing reactions need to be considered carefully and will probably include liver function test results. However, we have not yet assessed the incidence of viral hepatitis in DART, and therefore its potential contribution to the hepatotoxicity observed is unknown. As expected, there were more LFT abnormalities in the nevirapine group: the clinical significance of the small differences in other laboratory parameters is unclear.

Limitations of our study include the fact that the toxicity rates in NORA were lower than originally anticipated, and therefore the study was slightly underpowered to determine the significance of the 50% reduction observed. The contribution of pharmacogenomics to the lower than anticipated toxicity rate is unknown. The NORA population is likely to differ substantially from previously studied groups in major MHC alleles such as HLA-B*5701 which may play a role in HSR (Hughes *et al.* 2004; Martin *et al.* 2006); a study of polymorhpisms in NORA is underway. Of note, one participant with an abacavir reaction was incorrectly switched to open label nevirapine after unblinding and experienced a nevirapine reaction 4 weeks later, suggesting that underlying susceptibility to HSR may be important. Two participants with suspected HSRs on blinded nevirapine subsequently continued open-label drug. It is therefore possible that more nevirapine reactions could have been treated through were the participants not part of a blinded study, although this does not affect the estimates of the rates of such SARs. Finally, DART does not include prospective HIV RNA viral load data, so the virological efficacy of abacavir *vs.* nevirapine in this population is not known, although triple NRTI regimens have had poorer virological efficacy in previous studies (Gulick *et al.* 2004). These tests are now being performed retrospectively.

In summary, in African patients initiating ARVs with low CD4 counts, there was a lower discontinuation rate of abacavir and a trend to a lower rate of SARs compared with nevirapine. With the exception of hepatotoxicity, there was considerable overlap in the clinical reactions to abacavir and nevirapine in this population. We estimate the rate of abacavir-HSR to be 2% in Uganda. Assessment of genetic polymorphisms that may, at least in part, explain why the event rate is lower than observed in industrialized countries is ongoing. These data suggest that the potential for abacavir HSR should not prevent its use in rollout as one of the recommended first-line NRTI options in the most recent WHO treatment guidelines (WHO 2006).
